# The role of psychological stress in the subjective well-being of aviation ground crews: mediating effects of social support and self-esteem

**DOI:** 10.1186/s12889-025-24406-4

**Published:** 2025-09-01

**Authors:** Qin Zhai, Taiyu Liu, Jiafei Bao, Yan Li, Xin Ji, Jianquan Tian

**Affiliations:** 1 Naval Medical Center, Naval Medical University, Shanghai, China; 2 The Third Affiliated Hospital of Naval Medical University, Naval Medical University, Shanghai, China; 3 Psychiatric Center of PLA, No.904 Hospital of The PLA Joint Logistic Support Force, Changzhou, China

**Keywords:** Ground crew, Psychological stress, Subjective well-being, Social support, Self-esteem

## Abstract

**Background:**

This study investigated the relationship between psychological stress and subjective well-being among aviation ground crews, with a focus on the mediating roles of social support and self-esteem.

**Methods:**

In this cross-sectional investigation, 533 ground crew members completed validated assessments, including the Stress Self-evaluation Test, the Perceived Social Support Scale, the Self-liking/Self-competence Scale-Revised, the Satisfaction with Life Scale and the Positive and Negative Affect Scale. Statistical analyses were performed via R software and Mplus.

**Results:**

(1) Single participants presented significantly lower subjective well-being than married individuals did (*p* = 0.001, Cohen’s *d* = 0.349). (2) Psychological stress (*r* = – 0.527, *p* < 0.001), social support (*r* = 0.579, *p* < 0.001), and self-esteem (*r* = 0.678, *p* < 0.001) were significantly correlated with subjective well-being. (3) Three distinct mediation pathways emerged: the social support-mediated pathway, self-esteem-mediated pathway, and a chain-mediating effect through social support-self-esteem.

**Conclusions:**

Psychological stress was significantly associated with reduced subjective well-being in aviation ground crews, with tentative evidence of mediation through social support and self-esteem. Interventions targeting these psychosocial resources may improve well-being, but require further validation.

**Supplementary Information:**

The online version contains supplementary material available at 10.1186/s12889-025-24406-4.

## Introduction

Aviation constitutes an indispensable component of contemporary multimodal transportation systems, serving as a pivotal modality for global socioeconomic connectivity and logistical optimization. Aviation ground services, an indispensable operational component of this industry, directly impact flight operation efficiency and airworthiness assurance. The ground crew, as the primary workforce responsible for preflight technical verification and maintenance of aircraft airworthiness, encompasses core technical positions, including but not limited to cargo handlers, fuel technicians, water/toilet system operators, catering support staff, de-icing specialists, aviation inspectors, and maintenance technicians [1]. These roles constitute the critical safety barrier against aviation incidents. However, their occupational environment—characterized by prolonged exposure to airport taxiways, ramps, and aprons—presents multifaceted hazards. Chronic biomechanical stress from sustained nonneutral postures (e.g., cervical hyperextension and lumbar flexion), shift work-induced circadian disruption, and direct dermal/olfactory contact with aviation turbine fuels and lubricants have been documented to induce cumulative physiological and psychological strain [2–4]. High-intensity task demands, coupled with perfectionism-driven workflows, exacerbate work‒life boundary permeation—a phenomenon associated with increased psychophysiological burden [5]. Operational stressors manifest as persistent cognitive hypervigilance, task-related anxiety, and fatigue-related performance degradation, compromising individual well-being and posing systemic risks to aviation safety outcomes [6]. Despite their pivotal role in aviation safety, ground crews remain an understudied population in occupational mental health research. Existing literature has focused predominantly on pilots [7], leaving a critical gap in understanding how their specific stressors impact well-being—and by extension, safety-critical performance.

The conceptualization of well-being has evolved toward subjective evaluations, formally termed “subjective well-being” [8]. Grounded in Diener’s definition [9, 10], subjective well-being comprises two core components: a cognitive dimension reflecting global judgments of life satisfaction, and an affective dimension characterized by the predominance of positive affect over negative affect. This construct is particularly salient in high-stress occupational cohorts such as aviation industry personnel, where targeted well-being interventions demonstrate efficacy in enhancing work-related quality of life and advancing management practices [11]. Crucially, subjective well-being exhibits stronger associations with modifiable psychological resilience factors (e.g., self-esteem, social support) than sociodemographic characteristics (e.g., income, occupation, educational attainment) [10, 12–16], justifying our investigation into these specific mediators.

Psychological stress occurs when environmental demands exceed an individual’s adaptive capacity [17]. Research has focused predominantly on stress responses reflecting such overload, including negative emotions, cognition, and behaviours triggered by inadequate coping resources during threatening scenarios [18, 19]. Psychological stress inversely predicts subjective well-being by depleting psychological energy, threatening self-worth, and amplifying negative affect while suppressing positive emotions [20, 21]. This association emerges as early as preadolescence, serving as a longitudinal predictor of subjective well-being trajectories [22]. Major life events, exemplified by the COVID-19 pandemic—a global crisis inducing unprecedented health and economic disruptions—represent critical environmental stressors. Inadequate stress management during such events directly compromises subjective well-being [23, 24]. Social support significantly mitigated the impacts of COVID-19-related stress during isolation periods [25]. Furthermore, elevated self-esteem reduces stress perception through adaptive cognitive appraisals, thereby preventing stress-related internalizing symptoms [26].

The stress process model necessitates the concurrent examination of personal resources (e.g., self-esteem) and social resources (e.g., social support) in buffering or moderating stressors. Stressor exposure may activate proactive social support-seeking behaviours and self-esteem-driven coping strategies [27]. For individuals with low self-esteem, stressors exert detrimental effects regardless of social support availability. Conversely, among high self-esteem individuals, stressor impacts vary with perceived social support levels, indicating that social support’s moderating effects on stressor‒outcome relationships are contingent upon baseline self-esteem [28]. A longitudinal study demonstrated the self-esteem antecedent model, wherein self-esteem predicts subsequent changes in perceived social support quality and network size [29]. In contrast, evidence suggests that increased social support may increase self-esteem, fostering adaptive coping strategies and positive stress appraisals that mitigate stressor impacts and facilitate post-stress growth [30]. Overall, optimal self-esteem levels and adequate social support serve as critical protective factors that attenuate stress-induced impairments in subjective well-being.

Although the detrimental effects of psychological stress on subjective well-being have been extensively documented in general populations, few studies have investigated the underlying mechanisms of this association among ground crews, particularly with respect to dual mediation pathways involving self-esteem and social support. Aviation ground crews represent a high-priority cohort for public health intervention, and their unique stressors (e.g., safety-critical task pressure, environmental hazards) may alter the underlying mechanisms of this association. In addition, the dual mediating roles of social support and self-esteem have rarely been tested in high-risk occupational contexts, where external support networks and internal resilience may interact differently than in general populations. As frontline safety personnel, ground crews’ well-being directly impacts aviation security. Understanding whether resource-building interventions (e.g., social support networks, esteem-cultivating training) can disrupt stress-subjective well-being pathways in this cohort holds direct implications for reducing turnover and enhancing occupational health equity in “invisible” essential workforce. To address this gap, we propose the following:

### Hypothesis 1

Psychological stress negatively predicts subjective well-being.

### Hypothesis 2

Social support mediates the relationship between psychological stress and subjective well-being;

### Hypothesis 3

Self-esteem mediates the relationship between psychological stress and subjective well-being;

### Hypothesis 4

The relationship between psychological stress and subjective well-being is serially mediated through social support and self-esteem.

## Methods

### Participants and procedure

A priori sample size calculation was performed via G*Power 3.1.9.6 [31, 32]. On the basis of a two-tailed independent t test with an effect size of d = 0.5 (medium effect size) [33], α = 0.05, and statistical power (1–β) = 0.90, the analysis indicated a required sample size of 140 participants (70 per group). To account for potential incomplete responses, the target sample was increased by 20% (final *n* = 175).

This cross-sectional study was conducted between September and December 2021 at two major airports in Liaoning Province, Northeast China. These airports were strategically selected for their pivotal roles in Northeast China’s aviation network, and they experience the most severe de-icing demands in the region—operational stressors directly relevant to our research focus. According to a study by Dong [34], and the global survey on gender in aviation released by the International Civil Aviation Organization in 2023 [35], male technicians constitute over 93% of the workforce in these roles. The lack of female participants mirrors the current gender distribution in Chinese technical ground handling positions. Therefore, the study focused exclusively on male aviation ground crews, defined as frontline technicians directly involved in aircraft inspection, avionics maintenance, and airworthiness certification. The inclusion criteria required at least three months of continuous service in technical positions, with the exclusion of temporary or contract workers and personnel in special physiological status. The target population was identified through airport human resource registries, which provided a comprehensive list of all personnel meeting the inclusion criteria. A census sampling method was employed, whereby all eligible individuals were invited to participate, minimizing selection bias by avoiding arbitrary exclusion of any qualified participants. This approach ensured that the sample reflected the full spectrum of eligible frontline technicians at the two airports. All participants independently completed the questionnaire in a designated room at the airport. Throughout the survey administration, trained investigators with comprehensive expertise in all questionnaire components provided assistance exclusively when participants requested clarification regarding the survey instruments.

The participants were requested to complete a paper-based questionnaire encompassing sociodemographic variables (i.e., age, marital status, and singleton status), stress perception, social support, self-esteem, and subjective well-being. Written informed consent was obtained from all participants prior to their enrolment in the study. The anonymity and confidentiality of the collected data were rigorously maintained throughout the research process. No financial compensation or material incentives were offered for participation in this investigation. The study procedures were conducted in accordance with the Declaration of Helsinki. The study protocol was approved by the Ethics Review Committee at the authors’ hospital.

Of the 548 eligible participants invited, 533 completed valid questionnaires, resulting in a 97.26% response rate. This high response rate further minimizes non-response bias, enhancing the reliability of the sample. The final sample (*n* = 533) far exceeds the a priori sample size requirement (*n* = 175) calculated via G*Power, ensuring sufficient statistical power to detect the hypothesized effects. The study cohort comprised exclusively male participants (*N* = 533) due to the absence of female representation, with a mean age of 24.89 years (SD = 3.61). The demographic characteristics revealed that 455 individuals (85.37%) reported being single, whereas 221 participants (41.46%) identified as only children within their respective families.

### Measures

The Chinese version of the stress self-evaluation test (SSET) was utilized to assess subjective psychological stress levels. This 10-item scale measures stress symptoms over the past 2 weeks. (e.g., “I remain vigilant towards dangerous situations”), has been previously validated in Chinese populations, with established reliability (Cronbach’s α = 0.82, McDonald’s ω = 0.85 in the current study) [36]. The participants rated items on a three-point Likert scale (1 = never; 3 = always). The raw score is calculated by summing the scores of all individual items, which are then statistically standardized into T scores ( $T=50+10( \chi -\bar{ \chi }/S)$, where χ denotes the raw score, $\bar{ \chi }$ represents the mean score, and *S* indicates the standard deviation). Higher T scores indicate a greater level of psychological stress experienced by the individual.

The 12-item Perceived Social Support Scale, whose Chinese version has been validated in prior studies with Chinese samples, was adopted to evaluate social support from family, friends, and other means [37, 38]. In the current study, it demonstrated good reliability (Cronbach’s α = 0.94, McDonald’s ω = 0.95). The response options ranged from 1 (very strongly disagree) to 7 (very strongly agree). The scores of each item were summed to obtain the total score. Higher scores indicate a greater amount of social support available to the individual.

The Self-liking/Self-competence Scale-Revised (SLCS-R), employed to capture global self-esteem, has a validated Chinese version with established psychometric properties in Chinese populations [39, 40]. Grounded in two-dimensional self-esteem theory, it consists of two subscales, each containing eight items. Sample items include “I tend to devalue myself” for the self-liking subscale and “I am highly effective at the things I do” for the self-competence subscale. In the current study, both subscales showed good reliability (self-liking: Cronbach’s α = 0.86, McDonald’s ω = 0.91; self-competence: Cronbach’s α = 0.83, McDonald’s ω = 0.87). Items were rated on a five-point scale (1 = strongly agree to 5 = strongly disagree), with higher scores indicating higher levels of self-esteem.

Subjective well-being was assessed using a composite measure consistent with its classic conceptualization—encompassing life satisfaction, positive affect, and negative affect [41]. This subjective well-being measure includes three subscales: (1) the Satisfaction with Life Scale (SWLS) comprises five items (e.g., “The conditions of my life are excellent”) rated on a seven-point agreement scale (1 = strongly disagree to 7 = strongly agree), (2) a 6-item positive affect subscale (e.g., “happy”) rated on a 7-point frequency scale (1 = not at all to 7 = extremely much), (3) a 6-item negative affect subscale (e.g., “sad”) rated on a 7-point frequency scale (1 = not at all to 7 = extremely much). The total 17-item structure and scoring follow the validated approach [42], which reported good psychometric properties for the composite index in Chinese populations (Cronbach’s α = 0.86, KMO = 0.85, factor loadings > 0.5). In the current study, the composite measure demonstrated good reliability (Cronbach’s α = 0.88, McDonald’s ω = 0.92). The composite subjective well-being index in the current study was calculated by summing the total score of SWLS, the positive affect score, and the reverse-scored negative affect score, consistent with the validated approach and justified by the conceptual equivalence of the three components in defining subjective well-being [9, 10, 41, 42]. Higher total scores indicate greater perceived well-being.

### Statistical analysis

Statistical analyses were performed via R software (Version 4.2.1) and Mplus (Version 8.3) [43, 44].

Prior to data analysis, Harman’s single-factor test was performed to assess common method bias. To summarize sample characteristics, descriptive statistics (means, standard deviations, and frequencies) were computed via the “psych” package (Version 2.4.6) in R. Reliability was assessed through both Cronbach’s α coefficient and McDonald’s ω coefficient [45, 46], where values exceeding 0.70 for Cronbach’s α coefficient and values above 0.80 for McDonald’s ω coefficient were considered indicative of robust internal consistency [19, 47]. A bivariate Pearson’s correlation analysis was conducted to examine the interrelationships among the variables of psychological stress, self-esteem, social support, and subjective well-being. Independent-samples t tests were implemented via the “rstatix” package (Version 0.7.2) to examine the mean differences in scale scores between marital status groups (single vs. married) and sibling composition groups (only child vs. non-only child) [48].

To elucidate the specific mediating pathways of social support and self-esteem in the relationship between psychological stress and subjective well-being, structural equation modelling (SEM) was implemented via Mplus (version 8.3) [44]. Latent variables (psychological stress, self-esteem, social support, and subjective well-being) were constructed via item parcelling to reduce measurement error and enhance model parsimony. Following Wu and Wen [49], the items for each latent construct were randomly assigned to three or more balanced parcels. Confirmatory factor analysis (CFA) was performed to validate the measurement model, with model fit evaluated via the following criteria: ratio of chi-square values to degrees of freedom (chi-square/DF) < 3 [50], comparative fit index (CFI) ≥ 0.90, Tucker‒Lewis index (TLI) ≥ 0.90 [51], root mean square error of approximation (RMSEA) ≤ 0.08 [52], and standardized root mean square residual (SRMR) ≤ 0.08 [53]. All the retained factor loadings exceeded 0.60, ensuring adequate construct reliability [54]. The hypothesized multiple mediation model was tested against three competing models: (1) a direct effect model (no mediators); (2) a single mediator model including only social support; (3) a single mediator model including only self-esteem. No post-hoc modifications were made to avoid overfitting. Model comparisons used rigorous fit indices where |ΔCFI| >0.01 indicates significant deterioration [53, 55] The model parameters were estimated via robust maximum likelihood estimation (MLR) for both the estimation and the mediation analyses. Bootstrap resampling (1000 iterations) was employed to derive bias-corrected 95% confidence intervals (CIs) for indirect effects, which enhances statistical power and minimizes Type I error in mediation analysis [56].

## Results

A Harman’s single-factor test was conducted to address potential common method bias. The unrotated exploratory factor analysis revealed that the first factor accounted for 28.6% of the total variance, falling below the critical threshold of 40% recommended for substantial common method variance [57]. These results do not suggest severe common method bias in the current dataset.

Assessment of psychological variables stratified by marital status revealed significantly lower subjective well-being in single participants versus married (*p* = 0.001, Cohen’s *d* = 0.349). No significant associations emerged for singleton status across all psychological measures: stress (*p* = 0.638, *d* = -0.041), social support (*p* = 0.322, *d* = -0.088), self-esteem (*p* = 0.653, *d* = 0.040), and subjective well-being (*p* = 0.957, *d* = 0.005) (Table 1).

**Table 1:** Comparison of participants’ stress, social support, self-esteem and subjective well-being scores according to demographic variables

Demographic variables	n	Stress			Social support			Self-esteem			Subjective well-being		
		Mean ± SD	*t*, *p*	Effect size	Mean ± SD	*t*, *p*	Effect size	Mean ± SD	*t*, *p*	Effect size	Mean ± SD	*t*, *p*	Effect size
Single	78	50.02 ± 10.09	*t* = – 0.015 *p* = 0.988	*d* = – 0.002	67.68 ± 11.40	*t* = – 1.006 *p* = 0.316	*d* = – 0.107	59.22 ± 9.24	*t* = – 0.397 *p* = 0.692	*d* = – 0.049	91.03 ± 14.65	*t* = – 3.270 *p* = 0.001	*d* = – 0.349
Married	455	50.04 ± 9.27			68.87 ± 9.30			59.67 ± 8.83			96.03 ± 12.06		
Only child	221	50.27 ± 10.21	*t* = 0.470 *p* = 0.638	*d* = 0.041	68.43 ± 11.49	*t* = 1.000 *p* = 0.318	*d* = 0.088	59.07 ± 9.62	*t* = – 0.450 *p* = 0.653	*d* = – 0.040	91.72 ± 14.81	*t* = – 0.054 *p* = 0.957	*d* = – 0.005
Non-only child	312	49.85 ± 9.80			67.45 ± 10.85			59.44 ± 8.87			91.79 ± 14.13		

Pearson correlation analyses were conducted to examine the interrelationships among psychological stress, subjective well-being, social support, and self-esteem in ground crews. Descriptive statistics, such as skewness, kurtosis, means, and standard deviations, as well as bivariate correlations, are presented in Table 2. Psychological stress (*r* = – 0.527, *p* < 0.001), social support (*r* = 0.579, *p* < 0.001), and self-esteem (*r* = 0.678, *p* < 0.001) were significantly correlated with subjective well-being. Enhanced social support was positively associated with self-esteem (*r* = 0.577, *p* < 0.001). Elevated psychological stress was negatively correlated with both social support (*r* = – 0.444, *p* < 0.001) and self-esteem (*r* =-0.489, *p* < 0.001).

**Table 2:** Correlation coefficients for the stress, social support, self-esteem and subjective well-being variables

Variables	Descriptive statistics				Correlation coefficients			
	Skewness	Kurtosis	Mean	SD	1	2	3	4
1. Stress	0.892	0.729	50.02	9.96	1			
2. Social support	– 0.464	– 0.005	67.86	11.12	– 0.444^***^	1		
3. Self-esteem	– 0.058	– 0.103	59.29	9.18	– 0.489^***^	0.577^***^	1	
4. Subjective well-being	– 0.301	0.184	91.76	14.40	– 0.527^***^	0.579^***^	0.678^***^	1

^***^ *p* < 0.001

**Fig. 1:**
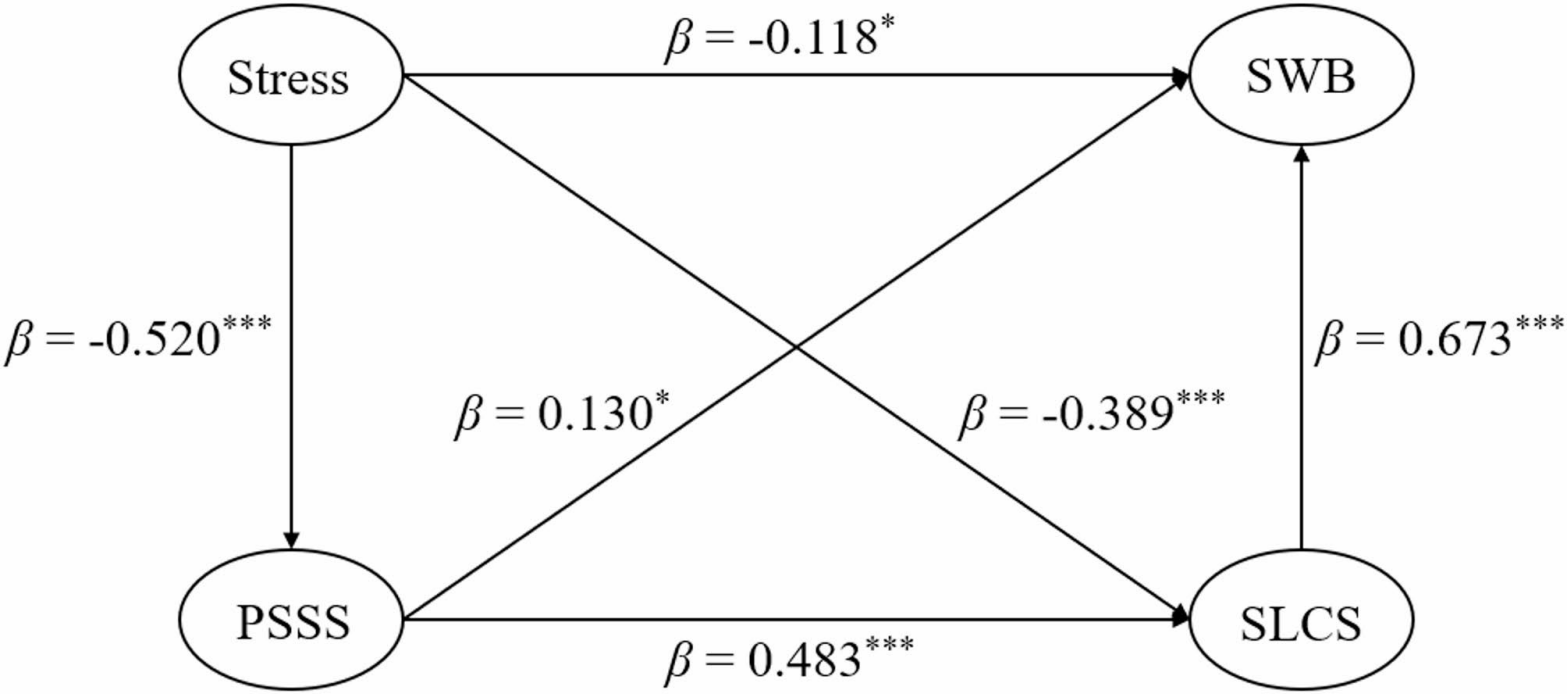
Multiple mediation model with standardized path coefficients. Values represent *β* coefficients. ^*^p < 0.05. ^***^ *p* < 0.001

Preliminary analyses indicated adequate model fit for the multiple mediation model (chi-square/DF = 175.852/84 = 2.093 < 3, RMSEA = 0.046, CFI = 0.983, TLI = 0.979, SRMR = 0.026; Fig. 1). Comparative model testing revealed no statistically significant differences between the hypothesized model and competing alternatives based on the ΔCFI > 0.01 threshold: (1) direct-effects model: chi-square/DF = 50.953/26 = 1.960 < 3, RMSEA = 0.043, CFI = 0.992, TLI = 0.988, SRMR = 0.019, |ΔCFI| = 0.009; (2) social support-mediation model: chi-square/DF = 99.737/51 = 1.956 < 3, RMSEA = 0.043, CFI = 0.989, TLI = 0.986, SRMR = 0.020, |ΔCFI| = 0.006; (3) self-esteem-mediation model: chi-square/DF = 117.707/51 = 2.308 < 3, RMSEA = 0.050, CFI = 0.982, TLI = 0.977, SRMR = 0.028, |ΔCFI| = 0.001. The hypothesized model was retained for its theoretical coherence with the proposed dual-mediation framework.

As detailed in Table 3, the mediation analysis revealed significant unstandardized effects across all pathways. The path diagram (Fig. 1) visually presents the standardized coefficients for these relationships, with all significant paths marked. Specifically, the diagram includes: (1) standardized direct effect of psychological stress on subjective well-being (*β* = – 0.118, *p* = 0.025, CI = [– 0.220, – 0.012]); (2) standardized paths from psychological stress to social support (*β* = – 0.520, *p* < 0.001, CI = [– 0.601, – 0.438]) and self-esteem (*β* = – 0.389, *p* < 0.001, CI = [– 0.491, – 0.291]); (3) standardized paths from social support to subjective well-being (*β* = 0.130, *p* = 0.026, CI = [0.014, 0.245]) and self-esteem (*β* = 0.483, *p* < 0.001, CI = [0.374, 0.580]); (4) standardized path from self-esteem to subjective well-being (*β* = 0.673, *p* < 0.001, CI = [0.535, 0.824]). These standardized coefficients in the path diagram complement the unstandardized effects in Table 3, providing a clear visual summary of the mediation model’s structure and significance.

**Table 3:** Unstandardized direct, indirect, and total effects among the variables in the model

Effect type	Path	Estimate	SE	*p*	Bootstrapping 95% CI	
					Lower 2.5%	Upper 2.5%
Total effect	Stress-SWB	– 2.539	0.270	< 0.001	– 3.112	– 2.065
Direct effect	Stress-SWB	– 0.485	0.223	0.030	– 0.904	– 0.048
*Indirect effects*						
Self-esteem-mediated	Stress-SLCS-SWB	– 1.079	0.205	< 0.001	– 1.538	– 0.732
Support-mediated	Stress-PSSS-SWB	– 0.279	0.130	0.032	– 0.544	– 0.030
Chain-mediated	Stress-PSSS-SLCS-SWB	– 0.696	0.135	< 0.001	– 1.008	– 0.467

Abbreviations: *PSSS* perceived social support score, *SE* standard error, *SLCS* self-liking/self-competence score, *SWB* subjective well-being score

## Discussion

 This study revealed significant disparities in subjective well-being across marital status groups, with psychological stress, social support, and self-esteem identified as critical determinants of subjective well-being. A negative correlation was observed between psychological stress and subjective well-being, indicating that elevated psychological stress levels in ground crews corresponded to diminished subjective well-being. Furthermore, a multiple mediation model was constructed to elucidate the mechanisms underlying the impact of psychological stress on subjective well-being. The findings demonstrated that psychological stress had both direct and indirect effects on subjective well-being. Notably, three distinct mediation pathways were identified: (1) social support-mediated pathway, (2) self-esteem-mediated pathway, and (3) a chain-mediating effect through social support-self-esteem. These results offer preliminary support for our theoretical hypotheses, suggesting that higher levels of self-esteem and social support may play a protective role in mitigating the negative impact of psychological stress on subjective well-being. However, the cross-sectional nature of the study necessitates caution in inferring causality; future longitudinal or intervention research is needed to confirm these mediating pathways and their causal direction.

 This study revealed significantly greater levels of subjective well-being among married participants than among their single counterparts, which aligns with existing evidence [58]. Compared with cohabitation, marital union confers more pronounced psychological benefits [59], primarily through mechanisms such as dyadic stress mitigation, shared responsibility in economic and domestic domains, and the provision of companionship and emotional support. These spousal resources enable married individuals to better navigate life challenges than their single counterparts (encompassing never-married, divorced, and widowed populations). However, the marriage–subjective well-being relationship may be influenced by a complex interplay of contextual factors across cultures, including socioeconomic status, parental status, marital duration, and residential patterns. Notably, the exclusively male sample from Northeast China limits demographic generalizability but reveals culturally-contextualized patterns. This gender homogeneity, while restricting demographic generalizability, provides a focused lens to examine masculine norms in Northeastern kinship systems. The observed marital status effect aligns with established mechanisms of marital resource pooling (e.g., dyadic stress buffering, economic cooperation). However, its magnitude may be amplified by Northeast China’s traditional kinship networks where multigenerational households intensify spousal support obligations. Concurrently, the high singleton prevalence (41.46%) corresponds to Liaoning Province’s historical implementation of China’s one-child policy, where only children face intensified achievement expectations. Future cross-cultural studies should examine the transferability of identified mediator pathways in societies with weaker familial collectivism.

 This study substantiates the hypothesized inverse relationship between psychological stress and subjective well-being, corroborating previous findings [60–62]. The mediation model revealed dual pathways: psychological stress directly predicts a reduction in subjective well-being while simultaneously exerting indirect effects through the social support and self-esteem pathways. In this study, psychological stress was operationalized as stress-induced symptomatology triggered by life events, encompassing physiological, psychological, and behavioural dysregulation. The key manifestations included hypervigilance, insomnia, emotional lability, fatigue, and anhedonia, all of which impair emotional regulation and cognitive appraisal, ultimately leading to a decline in subjective well-being [63]. Notably, individual responses to ubiquitous stressors vary significantly. While some individuals experience adverse outcomes, others demonstrate resilience or posttraumatic growth, which is attributable to protective external and internal factors. Social support emerged as a critical external buffer, operating through mechanisms outlined in the stress-buffering model [64]. Specifically, social support facilitates the cognitive reappraisal of stressors, provides practical assistance, reinforces companionship and mitigates the impact of stressors across diverse populations [65]. Løseth et al.‘s dyadic stress and support task results in accelerated stress recovery with social support, and Wang et al.‘s meta-analysis confirmed the protective role of social support against posttraumatic stress disorder (PTSD) symptoms [66, 67]. Notably, social support effectiveness depends on delivery modality, with invisible support providing more benefits than visible interventions do, as it avoids conveying sceptical attitudes during support provision [68]. Concurrently, self-esteem functions as a stable internal resilience factor. Neuroimaging evidence further indicates that low self-esteem increases stress–coping effort, depleting emotional–cognitive resources and ultimately exacerbating risks to health and well-being [69]. Individuals with high self-esteem exhibit an increased capacity to counteract stress through mindfulness and positive self-schema, particularly in digital social contexts [70]. This protective mechanism is generalizable across developmental stages, as evidenced by its stress-mitigating effects in adolescent populations in the region of Sfax-Tunisia [71].

 Our findings demonstrate the partial mediating role of self-esteem in the relationship between social support and subjective well-being. Grounded in social support resource theory [72], social support constitutes a critical external resource reservoir that interacts with intrinsic self-resources (e.g., self-esteem) to shape identity coherence and perceived environmental dependability [73]. This resource integration mechanism positions social support as a cornerstone of health and well-being. The developmental trajectory of the protective effects of self-esteem emerges during adolescence, as evidenced by Katsantonis et al.‘s data [74]. Specifically, self-esteem enhancement buffers childhood adversity survivors’ subjective well-being [75], whereas peer victimization-induced self-esteem reduction predicts compromised subjective well-being [76]. Empirical studies consistently demonstrate a reciprocal relationship between social support and self-esteem, wherein individuals with diminished social support accessibility exhibit compromised self-worth perceptions [77], whereas those with heightened self-esteem report increased social support availability [78], thereby establishing‌ a robust positive association between these psychological constructs [79–81].

Our findings tentatively suggest a mechanism whereby perceived social support, particularly from significant others, may influence individuals’ self-evaluations, serving as a form of relational validation that shapes personal value perception within social networks [82]. These sociocognitive processes appear to reconfigure self-perception and evaluative schemas, potentially positioning self-esteem as a bridge facilitating the translation of social support benefits into enhanced subjective well-being [83]. It is crucial to emphasize that these proposed pathways require robust confirmation through future research employing longitudinal designs and diverse samples to establish causality and generalizability.

While the identified mediation pathways involving social support and self-esteem offer plausible mechanisms linking psychological stress to subjective well-being, it is important to consider potential alternative explanations for the observed relationships. Other unmeasured or partially accounted for variables, such as dispositional personality traits (e.g., neuroticism, extraversion), habitual coping styles (e.g., positive vs. negative), and domain-specific factors like job satisfaction, could potentially confound or partially account for the associations reported. For instance, individuals higher in neuroticism may experience both greater perceived stress and lower subjective well-being [84, 85], while those employing positive coping strategies might report better well-being [86]. Similarly, job satisfaction could serve as a significant correlate of both lower stress appraisals and higher subjective well-being within this occupational context [87, 88]. Although our analysis controlled for key demographics andfocused on the hypothesised mediators, the cross-sectional design limits our ability to definitively rule out the influence of these or other third variables. Future research employing longitudinal designs and incorporating a broader range of psychological and contextual measures would be valuable to disentangle the unique contributions of the proposed mediators from these potential alternative influences on subjective well-being

 This investigation acknowledges these considerations regarding alternative explanations alongside four core methodological limitations. Foremost, the cross-sectional design inherently precludes causal inference regarding the stress-mediator-subjective well-being pathways observed. Second, while our male-exclusive Northeast China sample enabled deep cultural examination, it constrains generalizability to female aviation personnel and regions with divergent familial structures. Third, the dichotomous marital status metric oversimplified relational complexities; future research should incorporate relationship quality indicators. Finally, exclusive self-report reliance risks common-method bias; multimodal approaches integrating supervisor ratings or cortisol biomarkers would enhance objectivity. Collectively, these limitations underscore the preliminary nature of our findings regarding the mediating roles of social support and self-esteem. While the identified pathways offer promising directions, their confirmation and the development of effective, evidence-based interventions aimed at bolstering social support networks and enhancing self-esteem to improve subjective well-being among stressed populations necessitate rigorous future research, particularly longitudinal studies and randomized controlled trials.

## Conclusion

 This study identified psychological stress as a significant correlate of diminished subjective well-being among aviation ground crews. Our findings tentatively point to potential dual-path mediation mechanisms, where social support may foster external resources and self-esteem could bolster internal resilience, thereby extending our understanding of the complex interplay between stress, social support, self-esteem, and subjective well-being. These exploratory findings suggest that interventions focusing on enhancing social support networks and cultivating self-esteem might be beneficial pathways for improving subjective well-being in high-stress occupational groups like ground crews. However, the cross-sectional design precludes definitive causal claims. Future research, particularly longitudinal and intervention studies, is essential to rigorously confirm these mediating pathways and to develop and evaluate the effectiveness of targeted interventions aimed at mitigating stress impacts through these psychosocial resources for holistic well-being enhancement.

## Supplementary Information

Below is the link to the electronic supplementary material.

Supplementary Material 1

Supplementary Material 2

## Data Availability

The data that support the findings of this study are available from the corresponding author upon reasonable request.

## Abbreviations

CFA: Confirmatory factor analysis
CFI: Comparative fit index
|ΔCFI|: Change in comparative fit index
DF: Degrees of freedom
CI: Confidence interval
ICS: The International College Survey
MLR: Maximum likelihood estimation
PANAS: The positive and negative affect scale
PSSS: Perceived social support scores
PTSD: Post-Traumatic Stress Disorder
RMSEA: Root mean square error of approximation
SE: Standard error
SEM: Structural equation modelling
SLCS: Self-liking/self-competence scores
SLCS-R: The self-liking/self-competence scale-revised
SRMR: Standardized root mean square residual
SSET: The Chinese version of stress self-evaluation test
SWB: Subjective well-being scores
SWLS: The satisfaction with life scale
TLI: Tucker‒Lewis index
