# Supplementary material for: The role of psychological stress in the subjective well-being of aviation ground crews: mediating effects of social support and self-esteem
Source: BMC Public Health. 2025 Sep 1;25:2989. doi: 10.1186/s12889-025-24406-4 (PMC12400623; doi:10.1186/s12889-025-24406-4)
Supplement: Supplementary file 1 — Supplementary Material 1 [file 12889_2025_24406_MOESM1_ESM.docx]

**Supplementary Table 1** Full fit indices for competing models.

| Model Specification | chi-square (DF) | chi-square/DF | RMSEA | CFI | TLI | SRMR | \|ΔCFI\| |
| --- | --- | --- | --- | --- | --- | --- | --- |
| Hypothesized Model | 175.852(84) | 2.093 | 0.046 | 0.983 | 0.979 | 0.026 |  |
| Competing Model 1 (Direct-effects only) | 50.953(26) | 1.960 | 0.043 | 0.992 | 0.988 | 0.019 | 0.009 |
| Competing Model 2 (Single-mediation: Support only) | 99.737(51) | 1.956 | 0.043 | 0.989 | 0.986 | 0.020 | 0.006 |
| Competing Model 3 (Single-mediation: Self-esteem only) | 117.707(51) | 2.308 | 0.050 | 0.982 | 0.977 | 0.028 | 0.001 |

Abbreviations: CFA, confirmatory factor analysis; CFI, comparative fit index; DF , degrees of freedom; RMSEA, root mean square error of approximation; SRMR, standardized root mean square residual; TLI, Tucker-Lewis index; |ΔCFI|, change in comparative fit index.
