# Supplementary figures and images for: The role of psychological stress in the subjective well-being of aviation ground crews: mediating effects of social support and self-esteem
Source: BMC Public Health. 2025 Sep 1;25:2989. doi: 10.1186/s12889-025-24406-4 (PMC12400623; doi:10.1186/s12889-025-24406-4)

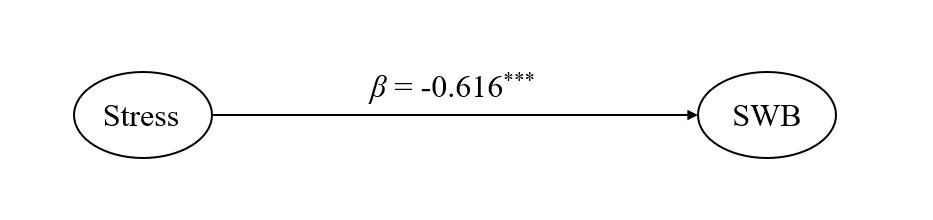

Supplement: Supplementary file 2 — Supplementary Material 2 [file 12889_2025_24406_MOESM2_ESM.zip › DIRECT.png]

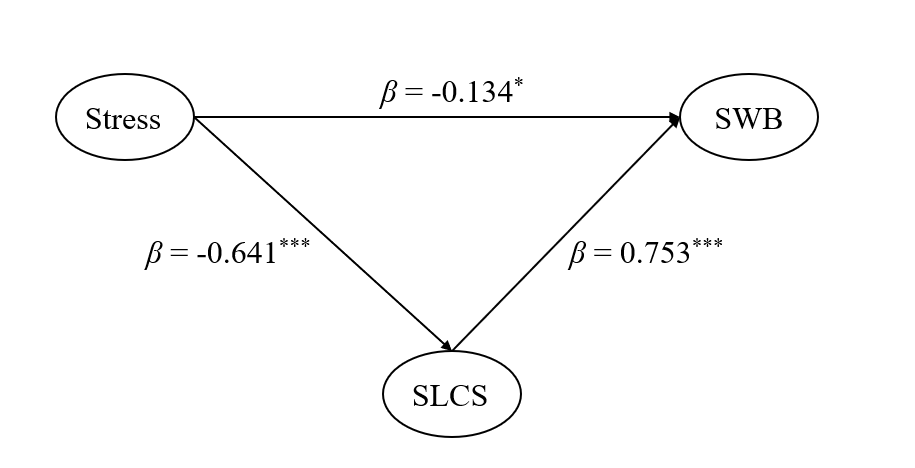

Supplement: Supplementary file 2 — Supplementary Material 2 [file 12889_2025_24406_MOESM2_ESM.zip › ESTEEM.png]

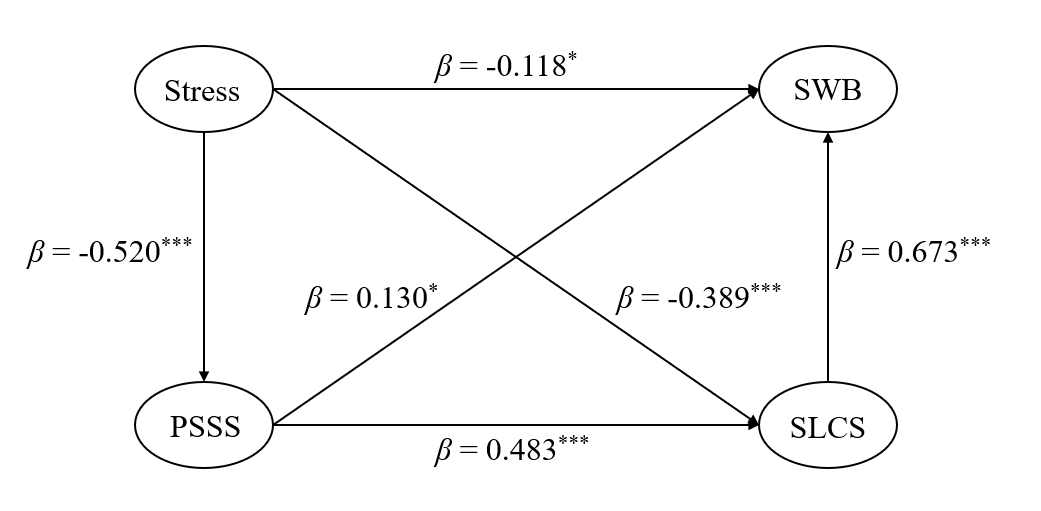

Supplement: Supplementary file 2 — Supplementary Material 2 [file 12889_2025_24406_MOESM2_ESM.zip › MEDIATION.png]

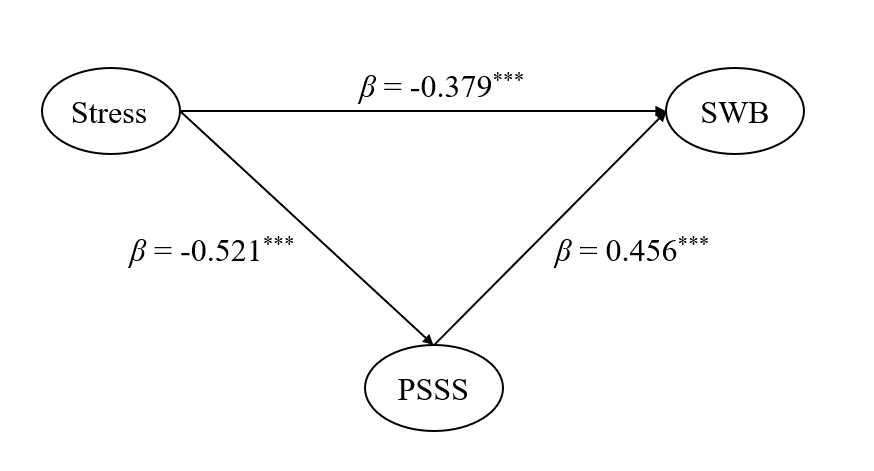

Supplement: Supplementary file 2 — Supplementary Material 2 [file 12889_2025_24406_MOESM2_ESM.zip › SUPPORT.png]
